# Cardiovascular Adverse Events Associated with Monoclonal Antibody Products in Patients with COVID-19

**DOI:** 10.3390/ph15121472

**Published:** 2022-11-26

**Authors:** Jingrui Zou, Fuyuan Jing

**Affiliations:** 1 Department of Scientific Affairs, Tongji Hospital, Tongji Medical College, Huazhong University of Science and Technology, Wuhan 430030, China; 2 Independent Researcher, Marlboro, NJ 07746, USA

**Keywords:** monoclonal antibodies, FAERS, cardiovascular adverse events, pharmacovigilance analysis

## Abstract

Little is known about cardiovascular safety profiles for monoclonal antibody products that received the FDA Emergency Use Authorization for COVID-19. In this study, data from the FDA Adverse Event Reporting System from the first quarter of 2020 to the second quarter of 2022 were used to investigate cardiovascular safety signals associated with seven monoclonal antibody products (casirivimab + imdevimab, bamlanivimab, bamlanivimab + etesevimab, sotrovimab, tocilizumab, bebtelovimab, tixagevimab + cilgavimab) in COVID-19 patients. Disproportionality analyses were conducted using reporting odds ratio and information component to identify safety signals. About 10% of adverse events in COVID-19 patients were cardiovascular adverse events. Four monoclonal antibody products (casirivimab + imdevimab, bamlanivimab, bamlanivimab + etesevimab, and bebtelovimab) were associated with higher reporting of hypertension. Tocilizumab was associated with higher reporting of cardiac failure and embolic and thrombotic event. Casirivimab + imdevimab and bamlanivimab were also associated with higher reporting of ischemic heart disease. No cardiovascular safety signals were identified for sotrovimab and tixagevimab + cilgavimab. The results indicate differential cardiovascular safety profiles in monoclonal antibodies. Careful monitoring of cardiovascular events may be considered for certain COVID-19 patients at risk when they are treated with monoclonal antibodies.

## 1. Introduction

As coronavirus disease 2019 (COVID-19) continues to be a global public health concern, various biologics have been developed or repurposed as a prophylactic or therapeutic strategy in the past number of years [1]. Among these, monoclonal antibodies have received increased attention and several products have received FDA Emergency Use Authorization (EUA) for COVID-19. Despite an emerging concern that not all of these antibodies are effective against the newest omicron variant [2,3], previous meta-analyses of efficacy studies have revealed that monoclonal antibodies for COVID-19 resulted in a lower rate of hospitalization or emergency department visit, a reduced mortality rate, or a decrease in development of clinical symptoms [4,5,6,7]. These studies also evaluated safety outcomes associated with the use of monoclonal antibodies for COVID-19 and it was concluded that these products were generally not associated with a significantly higher risk of adverse events [4,5,6,7]. It should be noted, however, that these meta-analyses were based on results from a limited number of randomized control trials, which may not necessarily focus on specific adverse events as their primary research objective, and they may not have a sufficient statistical power to detect rare adverse events.

Pharmacovigilance studies using real world data could be an appropriate approach to addressing the research question of the post-marketing safety surveillance for approved drugs. Of particular interest are cardiovascular adverse events possibly associated with the use of monoclonal antibodies for COVID-19 management. This interest is due to the following reasons. First, cardiovascular safety signals have been reported for other medications used to treat COVID-19, such as cardiac arrhythmias, torsade de points and QT prolongation associated with hydroxychloroquine/chloroquine [8,9,10] and cardiac arrest associated with remdesivir [11], but there is, to our knowledge, no published pharmacovigilance study on this topic for monoclonal antibodies for COVID-19. Second, cardiovascular safety of tocilizumab, a monoclonal antibody approved for treatment of rheumatoid arthritis, has been examined by a network meta-analysis; it was found that the number of major adverse cardiovascular events was higher for tocilizumab than rituximab from clinical trials in patients with rheumatoid arthritis [12]. Tocilizumab is one of the monoclonal antibody products that have received FDA EUA for COVID-19; nonetheless, there has been no published data specifically regarding cardiovascular events in COVID-19 patients, as also pointed out by a recent review on cardiotoxicity for pharmacotherapy of COVID-19 [13]. Moreover, it has been reported that patients with COVID-19 who used medications such as azithromycin had a higher risk of cardiac adverse events than patients without COVID-19 who used the same medication [14]. This indicates that COVID-19 itself may amplify cardiac risk for certain medications in the management of COVID-19. Taken together, there is an urgent need for pharmacovigilance research on the cardiovascular safety of monoclonal antibodies in patients with COVID-19 to address the evidence gap.

The objective of the study is to examine cardiovascular safety signals associated with seven monoclonal antibody products for management of COVID-19 using data from the FDA Adverse Event Reporting System (FAERS). The FAERS is a post-marketing safety surveillance database for adverse events of pharmaceutical products submitted to FDA by healthcare professionals, consumers and their representatives [15]. The information can be served as a data source for real-world surveillance of adverse events and can be used to identify safety signals, especially rare adverse events that may not be detected in premarketing studies or clinical trials [16]. There has been increased use of FAERS data for pharmacovigilance analysis in the past decade, due to its advantages in providing information on unknown but clinically relevant associations for clinical decision making, and for constructive suggestions for pharmaceutical companies about methods of pre-marketing data collection, given possible associations between pre-marketing data and post-marketing safety signals [17].

## 2. Results

### 2.1. Cardiovascular Adverse Events Reported by COVID-19 Patients

Table 1 lists the total number of adverse events and cardiovascular adverse events for all COVID-19 treatments and seven monoclonal antibody products, as well as characteristics of the patients such as age, gender, and body weight. From the first quarter of 2020 to the second quarter of 2022, a total of 47,327 adverse events were reported for treatment related to COVID-19. Among these reports, 4689 (9.9%) were cardiovascular adverse events. The percentage of cardiovascular adverse events among total adverse events reported varied among monoclonal antibody products. Sotrovimab had the lowest percentage of cardiovascular adverse events (5.7%) whereas tixagevimab + cilgavimab had the highest percentage of cardiovascular adverse events (13.0%). For all COVID-19 treatment, cardiac arrhythmia was the most frequently reported type of cardiovascular adverse event, accounting for 47% of cardiovascular adverse events reported or 4.7% of total adverse events. When examined by different types of monoclonal antibody products, hypertension was the most frequently reported type of cardiovascular adverse event for casirivimab + imdevimab, bamlanivimab, bamlanivimab + etesevimab, sotrovimab, and bebtelovimab, whereas embolic and thrombotic events were the most frequently reported type of cardiovascular adverse events for tocilizumab and tixagevimab + cilgavimab. The average age of patients was 58 years, their average body weight was 87 kg, and 52% of them were male. With regards to reporting source, consumers, physicians, pharmacists and other health professionals each reported about a quarter of the adverse events in the study. When examining these characteristics by different types of monoclonal antibody products, the average age of patients varied from 50 to 66 years, their average body weight varied from 73 to 93 kg, and the percentage of male patients ranged from 30% to 69%, whereas 67–94% of the data were reported by healthcare professionals.

### 2.2. Disproportionality Analysis

**Figure 1:**
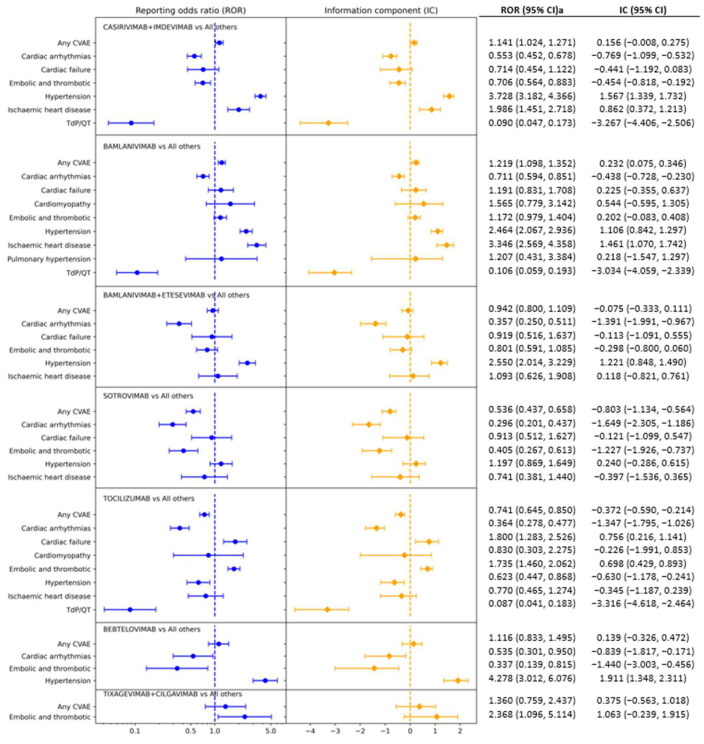
Forest plot of disproportionality analysis of cardiovascular adverse events (CVAE) for monoclonal antibody products for COVID-19. For each type of CVAE, only those with at least four reports were analyzed and presented. A significant safety signal is defined as the 95% confidence interval (CI) for ROR and is on the right side of the vertical reference line in blue (indicating that the lower bound of 95% CI is greater than 1) and the 95% CI for IC is on the right side of the vertical reference line in orange (indicating that the lower bound of 95% CI is greater than 0).

Figure 1 presents results from disproportionality analysis for each monoclonal antibody product for cardiovascular adverse events with at least four reports. Sample sizes used in the disproportionality analysis are presented in Supplementary Table S1. When both ROR and IC criteria were considered (the lower bound of 95% CI for ROR is greater than 1 and the lower bound of 95% CI for IC is greater than 0), the following cardiovascular safety signals were noted. Casirivimab + imdevimab was associated with hypertension (ROR = 3.728, 95% CI = (3.182, 4.366); IC = 1.567, 95% CI = (1.339, 1.732)) and ischemic heart disease (ROR = 1.986, 95% CI = (1.451, 2.718); IC = 0.862, 95% CI = (0.372, 1.213)). Bamlanivimab alone was associated with any cardiovascular adverse event (ROR = 1.219, 95% CI = (1.098, 1.352); IC = 0.232, 95% CI = (0.075, 0.346)), hypertension (ROR = 2.464, 95% CI = (2.067, 2.936); IC = 1.106, 95% CI = (0.842, 1.297)), and ischemic heart disease (ROR = 3.346, 95% CI = (2.569, 4.358); IC = 1.461, 95% CI = (1.070, 1.742)). Bamlanivimab + etesevimab was associated with hypertension (ROR = 2.550, 95% CI = (2.014, 3.229); IC = 1.221, 95% CI = (0.848, 1.490)). Tocilizumab was associated with cardiac failure (ROR = 1.800, 95% CI = (1.283, 2.526); IC = 0.756, 95% CI = (0.216, 1.141)) and embolic and thrombotic events (ROR = 1.735, 95% CI = (1.460, 2.062); IC = 0.698, 95% CI = (0.429, 0.893)). Bebtelovimab was associated with hypertension (ROR = 4.278, 95% CI = (3.012, 6.076); IC = 1.911, 95% CI = (1.348, 2.311)). No cardiovascular safety signal was detected for sotrovimab and tixagevimab + cilgavimab. Similar results were found from sensitivity analysis (Supplementary Figure S1) when analyses were restricted to data reported by healthcare professionals; the only exception is that the safety signal for cardiac failure associated with tocilizumab was no longer significant.

### 2.3. Outcomes Associated with Cardiovascular Adverse Events

Outcomes from cardiovascular adverse events and non-cardiovascular adverse events associated with use of each monoclonal antibody product for COVID-19 are presented in Table 2. Compared to non-cardiovascular adverse events, there was a higher percentage of patients with death as the outcome from cardiovascular adverse events associated with use of tocilizumab (49.8% vs. 37.0%, *p* < 0.001). Similarly, there was a higher percent of patients with life-threatening as the outcome from cardiovascular adverse events associated with use of tocilizumab (11.4% vs. 4.5%, *p* < 0.001). However, the percent of patients with hospitalization as the outcome from cardiovascular adverse events was lower than that from non-cardiovascular adverse events associated with use of tocilizumab (11.8% vs. 23.6%, *p* < 0.001). There was a higher percent of patients with hospitalization as the outcome from cardiovascular adverse events associated with use of casirivimab + imdevimab or bamlanivimab + etesevimab, compared to hospitalization from non-cardiovascular adverse events associated with the same monoclonal antibody product (51.4% vs. 41.8%, 49.6% vs. 35.2%, respectively, both *p* < 0.001). The percentage of patients reporting other serious important medical events as the outcome was lower in cardiovascular adverse events compared to non-cardiovascular adverse events associated with use of casirivimab + imdevimab (27.3% vs. 38.7%, *p* < 0.001). Comparisons of other outcomes or comparisons from other monoclonal antibody products were not statistically significant.

## 3. Discussion

Different cardiovascular safety signals for different monoclonal antibodies for COVID-19 were identified in the present study. For example, there is higher reporting of hypertension associated with use of casirivimab + imdevimab, bamlanivimab, bamlanivimab + etesevimab, and bebtelovimab for COVID-19. By contrast, there is a higher reporting of cardiac failure and embolic and thrombotic event associated with tocilizumab for COVID-19. Casirivimab + imdevimab and bamlanivimab for COVID-19 were also associated with higher reporting of ischemic heart disease as an adverse event. No safety signals were identified for sotrovimab and tixagevimab + cilgavimab. The results reveal differential safety profiles among various monoclonal antibodies and highlight the need for awareness of risk of certain cardiac events when treating COVID-19 patients with monoclonal antibodies.

The frequently found higher reporting of hypertension associated with several monoclonal antibodies requires further attention. While there have, to our knowledge, been no previous pharmacovigilance studies of monoclonal antibodies in COVID-19 patients from the published literature available for comparison with data from the present study, a randomized clinical trial evaluating the prophylactic effect of bamlanivimab on the incidence of COVID-19 reported hypertension to be the second-most common adverse event observed; nevertheless, there was no statistical difference when comparing the percentage of patients reporting hypertension as an adverse event following bamlanivimab administration with that in the placebo group [18]. Similarly, a retrospective study of 74 patients with COVID-19 reported hypertension to be one of the most common adverse events following tocilizumab administration based on new-onset hypertension noted in the medical records [19]. The causal relationship between monoclonal antibodies and hypertension in COVID-19 patients could not be established from published literature at this time, nonetheless, previous post-marketing safety studies of other monoclonal antibodies for the treatment of non-COVID-19 diseases have also reported similar results, such as the frequent report of hypertension as an adverse event for ramucirumab [20] and bevacizumab [21] in patients with colorectal cancer. Taken together, clinicians need to be aware of potential hypertension adverse events following monoclonal antibodies administration in patients with COVID-19 and consider blood pressure monitoring for patients at risk. This is particularly relevant since hypertension is also a common comorbidity in COVID-19 patients that is associated with a higher mortality rate [22].

While hypertension is one of the most commonly reported cardiovascular adverse events, different monoclonal antibodies for COVID-19 may also have a different cardiovascular safety profile as observed in the present study. Indeed, network meta-analysis of published clinical trials revealed a similar finding on the incidence of serious adverse events from different monoclonal antibodies for COVID-19, with sotrovimab ranking at the top in terms of the reduction in odds of serious adverse events [7]. In this study, that used real-world data, no cardiovascular safety signals were identified for sotrovimab and tixagevimab + cilgavimab. It should be noted that proportionality analysis on specific types of cardiovascular adverse event for tixagevimab + cilgavimab was limited due to the small number of cases available in the FAERS database.

Tocilizumab was associated with increased reporting of cardiac failure and embolic and thrombotic event in the present study. Recent clinical trials also reported cardiac arrhythmias [23] or embolic and thrombotic events [24] following tocilizumab administration in COVID-19 patients, although both studies reported a non-significant difference when comparing the percentage of these adverse events between tocilizumab with standard care and standard care alone. Higher reporting of ischemic heart disease was also found for casirivimab + imdevimab and bamlanivimab in the present study. Review of safety outcomes from published clinical trials of casirivimab + imdevimab and bamlanivimab [25,26,27,28,29,30,31,32] revealed that these studies did not report cardiovascular safety outcomes, or when detailed events were reported, ischemic heart disease was not among the list of adverse events found in their study populations. The inconsistent results from our pharmacovigilance analysis and safety outcomes from clinical trials may be partly explained by the lack of statistical power for rare adverse events in clinical trials.

The present study also compared outcomes of reported cardiovascular adverse events versus non-cardiovascular adverse events for each monoclonal antibody product. It was found that most of the outcomes were not significantly different between cardiovascular adverse events and non-cardiovascular adverse events. However, percentage of death and life-threatening outcomes for cardiovascular adverse events was higher than that for non-cardiovascular adverse events, for tocilizumab but not for other monoclonal antibody products. This may be because tocilizumab was authorized for hospitalized patients with severe COVID-19 condition, which are inherently associated with a higher risk for death or life-threatening outcomes, whereas other products were authorized for pre-exposure prophylaxis or treatment of mild to moderate COVID-19 conditions [33].

There are several limitations in the present study. First, the FAERS database consists of reported information from various sources; there can be a reporting bias as well as incomplete information in the report. Similar to other spontaneous reporting systems for adverse events for drugs, underreporting usually occurs due to reasons such as lack of awareness of reporting, uncertainty about causal effect, or simply because people may believe single case reported may not contribute to knowledge [34]. Second, a causal relationship could not be established from the disproportionality analysis. The adverse events reported may be caused by underlying diseases or comorbidities, other drugs, or unknown reasons. As such, it should be clearly noted that any significant safety signals identified from analysis of FAERS data does not necessarily mean that the drug caused the adverse event. To address this, well-designed clinical trials with sufficient sample sizes would be needed. Third, certain information such as comorbidities, COVID-19 disease severity, and baseline cardiovascular health status, is not available in the FAERS database. This information may contain important confounding factors to consider in future studies using other types of real-world data. Lastly, incidence of cardiovascular adverse events could not be estimated from the FAERS data given its nature. Despite these limitations, use of FAERS data for pharmacovigilance studies has been considered an effective approach to the identification of rare safety signals that might not necessarily be detected from clinical trials, and increases awareness of potential risks that can be examined by future studies or for consideration in clinical practice [17]. As the first pharmacovigilance study that examined cardiovascular safety associated with use of monoclonal antibodies products for COVID-19, our study highlights several cardiovascular safety signals that warrant further attention from manufacturers, regulatory agencies, healthcare professionals, and patients. It should be noted that results from the present study by no means discourage the use of monoclonal antibody products for COVID-19 treatments when following regulatory and clinical guidelines. Instead, it encourages future clinical studies to consider inclusion of cardiovascular adverse events as a secondary objective for future monoclonal antibody products being evaluated for COVID-19 management, as well as high-quality cohort studies evaluating the safety of novel monoclonal antibodies in a real-world setting.

## 4. Methods

### 4.1. Data Source

FAERS data were used in the study. Description of the data in the FAERS is available elsewhere [15]. Briefly, for each adverse event recorded in the database, the datasets include reporting source (the source where the event was reported from), demographic information (such as age, gender, event date, body weight, country), drug information (such as drug name, active ingredients, dose, form, route of administration, lot number, the role of the drug in the adverse event), therapy information (such as start and end of use date, duration), adverse events (coded adverse events), indications (coded indication for use for the reported drugs), and outcomes (patient outcomes for the adverse events) [15]. Adverse events and indications are coded as preferred terms according to the Medical Dictionary for Regulatory Activities (MedDRA). The database does not include market share information for a given medication. The database is updated quarterly and is publicly available, and the present study used FAERS data from the first quarter of 2020 to the second quarter of 2022, which corresponded to the start of the global COVID-19 pandemic and the latest dataset available at the time of the analysis in the study.

### 4.2. Study Population

The present study identified patients with COVID-19 using information from the “Indication” dataset and selected the following narrow standardized MedDRA query (SMQ) terms: COVID-19, COVID-19 pneumonia, Coronavirus infection, COVID-19 treatment, SARS-CoV-2 test positive, Suspected COVID-19, Corona virus infection, and Coronavirus test positive. Cases with these COVID-19-related preferred terms as indication were selected and records for these cases were extracted. Duplicated records were removed by keeping only the most recent version of the report from the same patient in the analysis. The present study was exempt from IRB review as publicly available, de-identified data were used.

### 4.3. COVID-19 Monoclonal Antibody Products

As of June 2022, monoclonal antibodies under FDA Emergency Use Authorization (EUA) include casirivimab + imdevimab, bamlanivimab + etesevimab, sotrovimab, tocilizumab, bebtelovimab, tixagevimab + cilgavimab [35]. Bamlanivimab alone also received FDA EUA in November 2020 but this was revoked in April 2021 due to increased frequency of resistant COVID-19 variants [36]. These seven monoclonal antibody products (casirivimab + imdevimab, bamlanivimab, bamlanivimab + etesevimab, sotrovimab, tocilizumab, bebtelovimab, tixagevimab + cilgavimab) were included for analysis in the present study. The drug name and active ingredient data in the “Drug” dataset were reviewed in order to identify records for these seven products that were either primary suspect or secondary suspect for the reported adverse event. Data for these records related to monoclonal antibodies were extracted and were then linked to the “Indication” dataset to identify records related to monoclonal antibodies used for COVID-19 treatment for subsequent analysis.

### 4.4. Cardiovascular Adverse Events

Cardiovascular adverse events from the “Reaction” dataset were identified using preferred terms falling into the following SMQ categories using the narrow scope of MedDRA (version 25.0): cardiac arrhythmias, cardiac failure, cardiomyopathy, embolic and thrombotic events, hypertension, ischemic heart disease, pulmonary hypertension, and Torsade de pointes/QT prolongation. This is similar to a previous study that comprehensively examined cardiovascular adverse events related to another medication using the FAERS dataset [37]. The list of preferred terms included in each of these eight categories can be found in Supplementary Table S2.

### 4.5. Other Data Extracted

Demographic information such as age, gender, and body weight; reporting source information; and outcome information (death, life-threatening, hospitalization, disability, congenital anomaly, required intervention to prevent permanent impairment, other serious medical events) were extracted from relevant datasets for identified cases. When multiple outcomes were reported for a case in the dataset, only the most severe outcome was counted in the analysis [38]. For example, if a patient had both hospitalization and death as outcomes recorded in the dataset, the patient was only counted for the death outcome in the analysis.

### 4.6. Safety Signal Detection

Cardiovascular safety signals were identified by disproportionality analyses using reporting odds ratio (ROR) and information component (IC). Both ROR and IC are valid methods for safety signal detection in pharmacovigilance studies [39,40] and have been widely used in pharmacovigilance studies with FAERS data [41,42]. ROR is estimated as the ratio for the odds of a cardiovascular adverse event reported for a monoclonal antibody in COVID-19 patients versus the odds of a cardiovascular adverse event reported for all other drugs in COVID-19 patients; a safety signal is detected if there are more than three adverse events of interest and the lower bound of the 95% confidence interval (CI) of ROR is greater than 1 [39,43]. IC is a measurement for strength of association between drugs and adverse events using the Bayesian neural network method; a safety signal is detected if the lower bound of the 95% CI of IC is greater than 0 [39,44]. The formulas used to calculate ROR and CI with their 95% CI are presented in Supplementary Table S3. In the present study, a safety signal is considered to be significant if both ROR and IC analyses have detected the same safety signal for the same drug [45,46]. A sensitivity analysis was conducted using only data reported from healthcare professionals (physicians, pharmacists, and health professionals).

### 4.7. Statistical Analysis

Data from relevant records in the FAERS dataset were imported into SQLite Studio (version 3.3.3, Hipp, Wyrick & Company, Inc., Charlotte, NC, USA) for processing. Statistical analysis was performed using Python (version 3.9.7, Python Software Foundation, Fredericksburg, VA, USA). Descriptive analysis was conducted to examine demographic characteristics and reporting source information. Data were presented as mean ± standard deviation for continuous variables or *n* (%) for categorical variables. Results from disproportionality analysis were presented as forest plots to facilitate visualization of safety signals. Outcomes from cardiovascular adverse events and non-cardiovascular adverse events associated with each monoclonal antibody product were compared by Chi-square test or Fishers exact test as appropriate; Bonferroni-corrected *p* < 0.05/49 = 0.001 was considered to be statistically significant.

## 5. Conclusions

In conclusion, differential cardiovascular safety profiles for monoclonal antibodies for COVID-19 were observed with pharmacovigilance analysis of FAERS data. Future clinical trials may consider adding cardiovascular safety as a secondary outcome. In clinical practice, careful monitoring of cardiovascular events, particularly blood pressure monitoring, may be considered for COVID-19 patients at risk when they are treated with monoclonal antibody administration.

## Figures and Tables

**Table 1:** Descriptive characteristics of cardiovascular adverse events (CVAE) reported for COVID-19 treatment and use of monoclonal antibody products.

	All COVID-19 Treatment	Casirivimab + Imdevimab	Bamlanivimab	Bamlanivimab + Etesevimab	Sotrovimab	Tocilizumab	Bebtelovimab	Tixagevimab + Cilgavimab
Total adverse events, *n*	47,327	3686	3797	1755	1765	3006	467	100
CVAE	4689 (9.9%)	407 (11.0%)	442 (11.6%)	165 (9.4%)	100 (5.7%)	230 (7.7%)	51 (10.9%)	13 (13.0%)
Cardiac arrhythmias	2215 (4.7%)	101 (2.7%)	131 (3.5%)	31 (1.8%)	26 (1.5%)	55 (1.8%)	12 (2.6%)	1 (1.0%)
Cardiac failure	351 (0.7%)	20 (0.5%)	33 (0.9%)	12 (0.7%)	12 (0.7%)	38 (1.3%)	0 (0.0%)	0 (0.0%)
Cardiomyopathy	75 (0.2%)	2 (0.1%)	9 (0.2%)	0 (0.0%)	0 (0.0%)	4 (0.1%)	0 (0.0%)	0 (0.0%)
Embolic and thrombotic events	1462 (3.1%)	83 (2.3%)	135 (3.6%)	44 (2.5%)	23 (1.3%)	151 (5.0%)	5 (1.1%)	7 (7.0%)
Hypertension	906 (1.9%)	210 (5.7%)	157 (4.1%)	79 (4.5%)	40 (2.3%)	37 (1.2%)	35 (7.5%)	3 (3.0%)
Ischemic heart disease	322 (0.7%)	46 (1.2%)	72 (1.9%)	13 (0.7%)	9 (0.5%)	16 (0.5%)	3 (0.6%)	3 (3.0%)
Pulmonary hypertension	42 (0.1%)	3 (0.1%)	4 (0.1%)	0 (0.0%)	0 (0.0%)	2 (0.1%)	0 (0.0%)	0 (0.0%)
Torsade de Pointes/QT prolongation	1168 (2.5%)	9 (0.2%)	11 (0.3%)	2 (0.1%)	0 (0.0%)	7 (0.2%)	1 (0.2%)	0 (0.0%)
Age reported, *n*	39,602	3549	3196	1557	1366	1611	390	92
Age, years, mean (SD)	58 (18)	54 (19)	66 (15)	52 (19)	50 (21)	59 (16)	52 (19)	58 (21)
Weight reported, *n*	17,527	2418	2320	1043	560	764	277	71
Weight, kg, mean (SD)	87 (28)	91 (27)	93 (26)	90 (28)	78 (26)	90 (26)	84 (24)	73 (23)
Gender reported, *n*	42,230	3583	3679	1678	1441	1788	448	96
Male	21,961 (52.0%)	1550 (43.3%)	1927 (52.4%)	664 (39.6%)	439 (30.5%)	1238 (69.2%)	146 (32.6%)	47 (49.0%)
Reporting sources, *n*	45,176	3127	3728	1508	1623	2967	362	86
Consumers	10,646 (23.6%)	353 (11.3%)	983 (26.4%)	254 (16.8%)	173 (10.7%)	183 (6.2%)	119 (32.9%)	12 (14.0%)
Physicians	9551 (21.1%)	256 (8.2%)	332 (8.9%)	161 (10.7%)	944 (58.2%)	1064 (35.9%)	22 (6.1%)	28 (32.6%)
Pharmacists	12,386 (27.4%)	1958 (62.6%)	1806 (48.4%)	859 (57.0%)	386 (23.8%)	625 (21.1%)	163 (45.0%)	39 (45.3%)
Other health professionals	12,593 (27.9%)	560 (17.9%)	607 (16.3%)	234 (15.5%)	120 (7.4%)	1095 (36.9%)	58 (16.0%)	7 (8.1%)

Data were presented as *n* (%) unless otherwise specified. SD: standard deviation.

**Table 2:** Outcomes from cardiovascular adverse events (CVAE) and non-CVAE associated with use of monoclonal antibody products for COVID-19.

	Casirivimab + Imdevimab	Bamlanivimab	Bamlanivimab + Etesevimab	Sotrovimab	Tocilizumab	Bebtelovimab	Tixagevimab + Cilgavimab
CVAE, *n*	370	383	141	86	229	46	13
Non-CVAE, *n*	2520	2111	1053	543	2199	266	69
Death							
CVAE, *n* (%)	28 (7.6%)	44 (11.5%)	12 (8.5%)	13 (15.1%)	114 (49.8%)	0 (0.0%)	2 (15.4%)
Non-CVAE, *n* (%)	102 (4.0%)	177 (8.4%)	61 (5.8%)	81 (14.9%)	813 (37.0%)	6 (2.3%)	4 (5.8%)
*p* value	0.002	0.049	0.206	0.962	**<0.001**	0.597	0.240
Life threatening							
CVAE, *n* (%)	28 (7.6%)	25 (6.5%)	12 (8.5%)	12 (14.0%)	26 (11.4%)	5 (10.9%)	1 (7.7%)
Non-CVAE, *n* (%)	116 (4.6%)	70 (3.3%)	92 (8.7%)	28 (5.2%)	99 (4.5%)	16 (6.0%)	11 (15.9%)
*p* value	0.014	0.003	0.929	0.002	**<0.001**	0.212	0.680
Hospitalization							
CVAE, *n* (%)	190 (51.4%)	237 (61.9%)	70 (49.6%)	28 (32.6%)	27 (11.8%)	15 (32.6%)	4 (30.8%)
Non-CVAE, *n* (%)	1053 (41.8%)	1330 (63.0%)	371 (35.2%)	205 (37.8%)	519 (23.6%)	49 (18.4%)	27 (39.1%)
*p* value	**<0.001**	0.676	**<0.001**	0.354	**<0.001**	0.028	0.757
Disability							
CVAE, *n* (%)	0 (0.0%)	0 (0.0%)	1 (0.7%)	1 (1.2%)	1 (0.4%)	0 (0.0%)	0 (0.0%)
Non-CVAE, *n* (%)	20 (0.8%)	5 (0.2%)	2 (0.2%)	5 (0.9%)	4 (0.2%)	2 (0.8%)	0 (0.0%)
*p* value	0.098	1.0	0.314	0.588	0.391	1.0	N/A
Congenital anomaly							
CVAE, *n* (%)	0 (0.0%)	0 (0.0%)	0 (0.0%)	0 (0.0%)	0 (0.0%)	0 (0.0%)	0 (0.0%)
Non-CVAE, *n* (%)	1 (0.0%)	0 (0.0%)	1 (0.1%)	0 (0.0%)	0 (0.0%)	0 (0.0%)	0 (0.0%)
*p* value	1.0	N/A	1.0	N/A	N/A	N/A	N/A
Required intervention to prevent permanent impairment/damage							
CVAE, *n* (%)	23 (6.2%)	1 (0.3%)	15 (10.6%)	2 (2.3%)	1 (0.4%)	5 (10.9%)	0 (0.0%)
Non-CVAE, *n* (%)	252 (10.0%)	8 (0.4%)	152 (14.4%)	44 (8.1%)	3 (0.1%)	57 (21.4%)	4 (5.8%)
*p* value	0.021	1.0	0.222	0.071	0.327	0.112	1.0
Other serious important medical event							
CVAE, *n* (%)	101 (27.3%)	76 (19.8%)	31 (22.0%)	30 (34.9%)	60 (26.2%)	21 (45.7%)	6 (46.2%)
Non-CVAE, *n* (%)	976 (38.7%)	521 (24.7%)	374 (35.5%)	180 (33.1%)	761 (34.6%)	136 (51.1%)	23 (33.3%)
*p* value	**<0.001**	0.041	0.001	0.751	0.011	0.493	0.528

Chi-square test or Fisher’s exact test was used to compare each outcome between CVAE and non-CVAE for each monoclonal antibody product. Bonferroni-corrected *p* < 0.05/49 = 0.001 was considered to be significant and was shown in bold.

## Data Availability

Data is contained within the article and supplementary materials.

## Supplementary Materials

The following supporting information can be downloaded at: https://www.mdpi.com/article/10.3390/ph15121472/s1, Table S1: Sample sizes used to calculate reporting odds ratio (ROR) and information component (IC) with their 95% confidence interval (CI) for disproportionality analysis; Table S2: Formulas used to calculate reporting odds ratio (ROR) and information component (IC) with their 95% confidence interval (CI) for disproportionality analysis; Table S3: Classification of preferred terms by standardized MedDRA query (SMQ) categories for cardiovascular adverse events; Figure S1: Sensitivity analysis: Forest plot of disproportionality analysis of cardiovascular adverse events (CVAE) for monoclonal antibody products for COVID-19, when only including CVAE reported by healthcare professionals. References [39,44,47] are cited in Supplementary Materials.
