# Supplementary material for: Cardiovascular Adverse Events Associated with Monoclonal Antibody Products in Patients with COVID-19
Source: Pharmaceuticals (Basel). 2022 Nov 26;15(12):1472. doi: 10.3390/ph15121472 (PMC9782649; doi:10.3390/ph15121472)
Supplement: Supplementary file 1 [file pharmaceuticals-15-01472-s001.zip › pharmaceuticals-1981720-supplementary.pdf]

Supplementary Table S1. Sample sizes used to calculate Reporting Odds Ratio (ROR) and Information Component (IC) with their 95% confidence interval (CI) for disproportionality analysis.

	# of CVAE for treatment	# of other events for treatment	# of CVAE for comparison	# of other events for comparison
**CASIRIVIMAB+IMDEVIMAB vs All others**				
Any CVAE	407	3279	4282	39359
Cardiac arrhythmias	101	3585	2114	41527
Cardiac failure	20	3666	331	43310
Embolic and thrombotic events	83	3603	1379	42262
Hypertension	210	3476	696	42945
Ischaemic heart disease	46	3640	276	43365
Torsade de pointes/QT prolongation	9	3677	1159	42482
**BAMLANIVIMAB vs All others**				
Any CVAE	442	3355	4247	39283
Cardiac arrhythmias	131	3666	2084	41446
Cardiac failure	33	3764	318	43212
Cardiomyopathy	9	3788	66	43464
Embolic and thrombotic events	135	3662	1327	42203
Hypertension	157	3640	749	42781
Ischaemic heart disease	72	3725	250	43280
Pulmonary hypertension	4	3793	38	43492
Torsade de pointes/QT prolongation	11	3786	1157	42373
**BAMLANIVIMAB+ETESEVIMAB vs All others**				
Any CVAE	165	1590	4524	41048
Cardiac arrhythmias	31	1724	2184	43388
Cardiac failure	12	1743	339	45233
Embolic and thrombotic events	44	1711	1418	44154
Hypertension	79	1676	827	44745
Ischaemic heart disease	13	1742	309	45263
**SOTROVIMAB vs All others**				
Any CVAE	100	1665	4589	40973
Cardiac arrhythmias	26	1739	2189	43373
Cardiac failure	12	1753	339	45223
Embolic and thrombotic events	23	1742	1439	44123
Hypertension	40	1725	866	44696
Ischaemic heart disease	9	1756	313	45249
**TOCILIZUMAB vs All others**				
Any CVAE	230	2776	4459	39862
Cardiac arrhythmias	55	2951	2160	42161
Cardiac failure	38	2968	313	44008
Cardiomyopathy	4	3002	71	44250

Embolic and thrombotic events	151	2855	1311	43010
Hypertension	37	2969	869	43452
Ischaemic heart disease	16	2990	306	44015
Torsade de pointes/QT prolongation	7	2999	1161	43160
**BEBTELOVIMAB vs All others**				
Any CVAE	51	416	4638	42222
Cardiac arrhythmias	12	455	2203	44657
Embolic and thrombotic events	5	462	1457	45403
Hypertension	35	432	871	45989
**TIXAGEVIMAB+CILGAVIMAB vs All others**				
Any CVAE	13	87	4676	42551
Embolic and thrombotic events	7	93	1455	45772

CVAE: cardiovascular adverse events

Supplementary Table S2. Classification of Preferred Terms by Standardized MedDRA Query (SMQ) categories for cardiovascular adverse events

SMQ categories	Preferred Terms
Cardiac arrhythmias	Chronotropic incompetence
Cardiac arrhythmias	Early repolarisation syndrome
Cardiac arrhythmias	Electrocardiogram repolarisation abnormality
Cardiac arrhythmias	Electrocardiogram RR interval prolonged
Cardiac arrhythmias	Electrocardiogram U wave inversion
Cardiac arrhythmias	Electrocardiogram U wave present
Cardiac arrhythmias	Electrocardiogram U-wave abnormality
Cardiac arrhythmias	Sudden cardiac death
Cardiac arrhythmias	Bradyarrhythmia
Cardiac arrhythmias	Ventricular asystole
Cardiac arrhythmias	Accessory cardiac pathway
Cardiac arrhythmias	Adams-Stokes syndrome
Cardiac arrhythmias	Agonal rhythm
Cardiac arrhythmias	Atrial conduction time prolongation
Cardiac arrhythmias	Atrial escape rhythm
Cardiac arrhythmias	Atrial standstill
Cardiac arrhythmias	Atrioventricular block
Cardiac arrhythmias	Atrioventricular block complete
Cardiac arrhythmias	Atrioventricular block first degree
Cardiac arrhythmias	Atrioventricular block second degree
Cardiac arrhythmias	Atrioventricular conduction time shortened
Cardiac arrhythmias	Atrioventricular dissociation
Cardiac arrhythmias	Atrioventricular node dysfunction
Cardiac arrhythmias	Bifascicular block
Cardiac arrhythmias	BRASH syndrome
Cardiac arrhythmias	Brugada syndrome
Cardiac arrhythmias	Bundle branch block
Cardiac arrhythmias	Bundle branch block bilateral
Cardiac arrhythmias	Bundle branch block left
Cardiac arrhythmias	Bundle branch block right
Cardiac arrhythmias	Conduction disorder
Cardiac arrhythmias	Defect conduction intraventricular
Cardiac arrhythmias	Electrocardiogram delta waves abnormal
Cardiac arrhythmias	Electrocardiogram PR prolongation
Cardiac arrhythmias	Electrocardiogram PR shortened
Cardiac arrhythmias	Electrocardiogram QRS complex prolonged
Cardiac arrhythmias	Electrocardiogram QT prolonged
Cardiac arrhythmias	Electrocardiogram repolarisation abnormality
Cardiac arrhythmias	Fascicular block
Cardiac arrhythmias	Lenegre's disease
Cardiac arrhythmias	Long QT syndrome
Cardiac arrhythmias	Paroxysmal atrioventricular block

Cardiac arrhythmias	Sinoatrial block
Cardiac arrhythmias	Trifascicular block
Cardiac arrhythmias	Ventricular dyssynchrony
Cardiac arrhythmias	Wolff-Parkinson-White syndrome
Cardiac arrhythmias	Atrial escape rhythm
Cardiac arrhythmias	Nodal arrhythmia
Cardiac arrhythmias	Nodal rhythm
Cardiac arrhythmias	Sinus arrest
Cardiac arrhythmias	Sinus arrhythmia
Cardiac arrhythmias	Sinus bradycardia
Cardiac arrhythmias	Sinus node dysfunction
Cardiac arrhythmias	Wandering pacemaker
Cardiac arrhythmias	Arrhythmia
Cardiac arrhythmias	Heart alternation
Cardiac arrhythmias	Heart rate irregular
Cardiac arrhythmias	Holiday heart syndrome
Cardiac arrhythmias	Pacemaker generated arrhythmia
Cardiac arrhythmias	Pacemaker syndrome
Cardiac arrhythmias	Paroxysmal arrhythmia
Cardiac arrhythmias	Pulseless electrical activity
Cardiac arrhythmias	Reperfusion arrhythmia
Cardiac arrhythmias	Withdrawal arrhythmia
Cardiac arrhythmias	Arrhythmia supraventricular
Cardiac arrhythmias	Atrial fibrillation
Cardiac arrhythmias	Atrial flutter
Cardiac arrhythmias	Atrial parasystole
Cardiac arrhythmias	Atrial tachycardia
Cardiac arrhythmias	Congenital supraventricular tachycardia
Cardiac arrhythmias	Frederick's syndrome
Cardiac arrhythmias	Junctional ectopic tachycardia
Cardiac arrhythmias	Sinus tachycardia
Cardiac arrhythmias	Supraventricular extrasystoles
Cardiac arrhythmias	Supraventricular tachyarrhythmia
Cardiac arrhythmias	Supraventricular tachycardia
Cardiac arrhythmias	Anomalous atrioventricular excitation
Cardiac arrhythmias	Cardiac fibrillation
Cardiac arrhythmias	Cardiac flutter
Cardiac arrhythmias	Extrasystoles
Cardiac arrhythmias	Tachyarrhythmia
Cardiac arrhythmias	Accelerated idioventricular rhythm
Cardiac arrhythmias	Arrhythmic storm
Cardiac arrhythmias	Cardiac fibrillation
Cardiac arrhythmias	Early repolarisation syndrome
Cardiac arrhythmias	Parasystole
Cardiac arrhythmias	Rhythm idioventricular
Cardiac arrhythmias	Torsade de pointes

Cardiac arrhythmias	Ventricular arrhythmia
Cardiac arrhythmias	Ventricular extrasystoles
Cardiac arrhythmias	Ventricular fibrillation
Cardiac arrhythmias	Ventricular flutter
Cardiac arrhythmias	Ventricular parasystole
Cardiac arrhythmias	Ventricular pre-excitation
Cardiac arrhythmias	Ventricular tachyarrhythmia
Cardiac arrhythmias	Ventricular tachycardia
Cardiac arrhythmias	Andersen-Tawil syndrome
Cardiac arrhythmias	Arrhythmia neonatal
Cardiac arrhythmias	Arrhythmogenic right ventricular dysplasia
Cardiac arrhythmias	Atrioventricular node dispersion
Cardiac arrhythmias	Brugada syndrome
Cardiac arrhythmias	Chronic atrial and intestinal dysrhythmia syndrome
Cardiac arrhythmias	Foetal arrhythmia
Cardiac arrhythmias	Foetal heart rate disorder
Cardiac arrhythmias	Foetal tachyarrhythmia
Cardiac arrhythmias	Heart block congenital
Cardiac arrhythmias	Junctional ectopic tachycardia
Cardiac arrhythmias	Long QT syndrome congenital
Cardiac arrhythmias	Lown-Ganong-Levine syndrome
Cardiac arrhythmias	Neonatal bradyarrhythmia
Cardiac arrhythmias	Neonatal tachyarrhythmia
Cardiac arrhythmias	Wolff-Parkinson-White syndrome congenital
Cardiac failure	Acute left ventricular failure
Cardiac failure	Acute pulmonary oedema
Cardiac failure	Acute right ventricular failure
Cardiac failure	Cardiac asthma
Cardiac failure	Cardiac failure
Cardiac failure	Cardiac failure acute
Cardiac failure	Cardiac failure chronic
Cardiac failure	Cardiac failure congestive
Cardiac failure	Cardiac failure high output
Cardiac failure	Cardiogenic shock
Cardiac failure	Cardiohepatic syndrome
Cardiac failure	Cardiopulmonary failure
Cardiac failure	Cardiorenal syndrome
Cardiac failure	Chronic left ventricular failure
Cardiac failure	Chronic right ventricular failure
Cardiac failure	Congestive hepatopathy
Cardiac failure	Cor pulmonale
Cardiac failure	Cor pulmonale acute
Cardiac failure	Cor pulmonale chronic
Cardiac failure	Ejection fraction decreased
Cardiac failure	Hepatojugular reflux

Cardiac failure	Left ventricular failure
Cardiac failure	Low cardiac output syndrome
Cardiac failure	Neonatal cardiac failure
Cardiac failure	Obstructive shock
Cardiac failure	Pulmonary oedema
Cardiac failure	Pulmonary oedema neonatal
Cardiac failure	Radiation associated cardiac failure
Cardiac failure	Right ventricular ejection fraction decreased
Cardiac failure	Right ventricular failure
Cardiac failure	Ventricular failure
Cardiomyopathy	Atrial septal defect acquired
Cardiomyopathy	Biopsy heart abnormal
Cardiomyopathy	Cardiac amyloidosis
Cardiomyopathy	Cardiac hypertrophy
Cardiomyopathy	Cardiac iron overload
Cardiomyopathy	Cardiac sarcoidosis
Cardiomyopathy	Cardiac septal hypertrophy
Cardiomyopathy	Cardiomyopathy
Cardiomyopathy	Cardiomyopathy acute
Cardiomyopathy	Cardiomyopathy alcoholic
Cardiomyopathy	Cardiomyopathy neonatal
Cardiomyopathy	Cardiotoxicity
Cardiomyopathy	Chagas' cardiomyopathy
Cardiomyopathy	Congestive cardiomyopathy
Cardiomyopathy	Diabetic cardiomyopathy
Cardiomyopathy	Ejection fraction abnormal
Cardiomyopathy	Ejection fraction decreased
Cardiomyopathy	Eosinophilic myocarditis
Cardiomyopathy	Giant cell myocarditis
Cardiomyopathy	HIV cardiomyopathy
Cardiomyopathy	Hypertensive cardiomyopathy
Cardiomyopathy	Hypertrophic cardiomyopathy
Cardiomyopathy	Ischaemic cardiomyopathy
Cardiomyopathy	Metabolic cardiomyopathy
Cardiomyopathy	Mitochondrial cardiomyopathy
Cardiomyopathy	Myocardial calcification
Cardiomyopathy	Myocardial fibrosis
Cardiomyopathy	Myocardial haemorrhage
Cardiomyopathy	Non-obstructive cardiomyopathy
Cardiomyopathy	Obesity cardiomyopathy
Cardiomyopathy	Pacing induced cardiomyopathy
Cardiomyopathy	Peripartum cardiomyopathy
Cardiomyopathy	Pulmonary arterial wedge pressure increased
Cardiomyopathy	Restrictive cardiomyopathy
Cardiomyopathy	Right ventricular ejection fraction decreased
Cardiomyopathy	Septic cardiomyopathy

Cardiomyopathy	Stress cardiomyopathy
Cardiomyopathy	Tachycardia induced cardiomyopathy
Cardiomyopathy	Thyrotoxic cardiomyopathy
Cardiomyopathy	Toxic cardiomyopathy
Cardiomyopathy	Ventricular septal defect acquired
Cardiomyopathy	Viral cardiomyopathy
Embolic and thrombotic events	Acute aortic syndrome
Embolic and thrombotic events	Acute coronary syndrome
Embolic and thrombotic events	Acute myocardial infarction
Embolic and thrombotic events	Amaurosis
Embolic and thrombotic events	Amaurosis fugax
Embolic and thrombotic events	Aneurysm thrombosis
Embolic and thrombotic events	Angioplasty
Embolic and thrombotic events	Aortic bypass
Embolic and thrombotic events	Aortic embolus
Embolic and thrombotic events	Aortic surgery
Embolic and thrombotic events	Aortic thrombosis
Embolic and thrombotic events	Aortogram abnormal
Embolic and thrombotic events	Arterectomy
Embolic and thrombotic events	Arterectomy with graft replacement
Embolic and thrombotic events	Arterial angioplasty
Embolic and thrombotic events	Arterial bypass operation
Embolic and thrombotic events	Arterial graft
Embolic and thrombotic events	Arterial occlusive disease
Embolic and thrombotic events	Arterial revascularisation
Embolic and thrombotic events	Arterial stent insertion
Embolic and thrombotic events	Arterial therapeutic procedure
Embolic and thrombotic events	Arterial thrombosis
Embolic and thrombotic events	Arteriogram abnormal
Embolic and thrombotic events	Arteriogram carotid abnormal
Embolic and thrombotic events	Arteriotomy
Embolic and thrombotic events	Atherectomy
Embolic and thrombotic events	Atherosclerotic plaque rupture
Embolic and thrombotic events	Atrial appendage closure
Embolic and thrombotic events	Atrial appendage resection
Embolic and thrombotic events	Basal ganglia infarction
Embolic and thrombotic events	Basilar artery occlusion
Embolic and thrombotic events	Basilar artery thrombosis
Embolic and thrombotic events	Blindness transient
Embolic and thrombotic events	Brachiocephalic artery occlusion
Embolic and thrombotic events	Capsular warning syndrome
Embolic and thrombotic events	Carotid angioplasty
Embolic and thrombotic events	Carotid arterial embolus
Embolic and thrombotic events	Carotid artery bypass
Embolic and thrombotic events	Carotid artery occlusion
Embolic and thrombotic events	Carotid artery stent insertion

Embolic and thrombotic events	Carotid artery thrombosis
Embolic and thrombotic events	Carotid endarterectomy
Embolic and thrombotic events	Cerebellar artery occlusion
Embolic and thrombotic events	Cerebellar artery thrombosis
Embolic and thrombotic events	Cerebral artery embolism
Embolic and thrombotic events	Cerebral artery occlusion
Embolic and thrombotic events	Cerebral artery stent insertion
Embolic and thrombotic events	Cerebral artery thrombosis
Embolic and thrombotic events	Cerebral hypoperfusion
Embolic and thrombotic events	Cerebrovascular insufficiency
Embolic and thrombotic events	Cerebrovascular stenosis
Embolic and thrombotic events	Coeliac artery occlusion
Embolic and thrombotic events	Coronary angioplasty
Embolic and thrombotic events	Coronary arterial stent insertion
Embolic and thrombotic events	Coronary artery bypass
Embolic and thrombotic events	Coronary artery embolism
Embolic and thrombotic events	Coronary artery occlusion
Embolic and thrombotic events	Coronary artery reocclusion
Embolic and thrombotic events	Coronary artery surgery
Embolic and thrombotic events	Coronary artery thrombosis
Embolic and thrombotic events	Coronary endarterectomy
Embolic and thrombotic events	Coronary revascularisation
Embolic and thrombotic events	Coronary vascular graft occlusion
Embolic and thrombotic events	Embolia cutis medicamentosa
Embolic and thrombotic events	Embolism arterial
Embolic and thrombotic events	Endarterectomy
Embolic and thrombotic events	Femoral artery embolism
Embolic and thrombotic events	Hepatic artery embolism
Embolic and thrombotic events	Hepatic artery occlusion
Embolic and thrombotic events	Hepatic artery thrombosis
Embolic and thrombotic events	Hypothenar hammer syndrome
Embolic and thrombotic events	Iliac artery embolism
Embolic and thrombotic events	Iliac artery occlusion
Embolic and thrombotic events	Internal capsule infarction
Embolic and thrombotic events	Intra-aortic balloon placement
Embolic and thrombotic events	Intraoperative cerebral artery occlusion
Embolic and thrombotic events	Ischaemic cerebral infarction
Embolic and thrombotic events	Ischaemic stroke
Embolic and thrombotic events	Lacunar infarction
Embolic and thrombotic events	Left atrial appendage closure implant
Embolic and thrombotic events	Leriche syndrome
Embolic and thrombotic events	Mesenteric arterial occlusion
Embolic and thrombotic events	Mesenteric arteriosclerosis
Embolic and thrombotic events	Mesenteric artery embolism
Embolic and thrombotic events	Mesenteric artery stenosis
Embolic and thrombotic events	Mesenteric artery stent insertion

Embolic and thrombotic events	Mesenteric artery thrombosis
Embolic and thrombotic events	Metabolic stroke
Embolic and thrombotic events	Myocardial infarction
Embolic and thrombotic events	Myocardial necrosis
Embolic and thrombotic events	Ophthalmic artery occlusion
Embolic and thrombotic events	Ophthalmic artery thrombosis
Embolic and thrombotic events	Papillary muscle infarction
Embolic and thrombotic events	Penile artery occlusion
Embolic and thrombotic events	Percutaneous coronary intervention
Embolic and thrombotic events	Peripheral arterial occlusive disease
Embolic and thrombotic events	Peripheral arterial reocclusion
Embolic and thrombotic events	Peripheral artery angioplasty
Embolic and thrombotic events	Peripheral artery bypass
Embolic and thrombotic events	Peripheral artery occlusion
Embolic and thrombotic events	Peripheral artery stent insertion
Embolic and thrombotic events	Peripheral artery surgery
Embolic and thrombotic events	Peripheral artery thrombosis
Embolic and thrombotic events	Peripheral embolism
Embolic and thrombotic events	Peripheral endarterectomy
Embolic and thrombotic events	Popliteal artery entrapment syndrome
Embolic and thrombotic events	Post procedural myocardial infarction
Embolic and thrombotic events	Postinfarction angina
Embolic and thrombotic events	Precerebral artery embolism
Embolic and thrombotic events	Precerebral artery occlusion
Embolic and thrombotic events	Precerebral artery thrombosis
Embolic and thrombotic events	Profundaplasty
Embolic and thrombotic events	Pseudo-occlusion of internal carotid artery
Embolic and thrombotic events	Pulmonary artery occlusion
Embolic and thrombotic events	Pulmonary artery therapeutic procedure
Embolic and thrombotic events	Pulmonary artery thrombosis
Embolic and thrombotic events	Pulmonary endarterectomy
Embolic and thrombotic events	Pulmonary tumour thrombotic microangiopathy
Embolic and thrombotic events	Renal artery angioplasty
Embolic and thrombotic events	Renal artery occlusion
Embolic and thrombotic events	Renal artery thrombosis
Embolic and thrombotic events	Renal embolism
Embolic and thrombotic events	Renal-limited thrombotic microangiopathy
Embolic and thrombotic events	Retinal artery embolism
Embolic and thrombotic events	Retinal artery occlusion
Embolic and thrombotic events	Retinal artery thrombosis
Embolic and thrombotic events	Segmental arterial mediolysis
Embolic and thrombotic events	Silent myocardial infarction
Embolic and thrombotic events	Spinal artery embolism
Embolic and thrombotic events	Spinal artery thrombosis
Embolic and thrombotic events	Splenic artery thrombosis
Embolic and thrombotic events	Splenic embolism

Embolic and thrombotic events	Stress cardiomyopathy
Embolic and thrombotic events	Subclavian artery embolism
Embolic and thrombotic events	Subclavian artery occlusion
Embolic and thrombotic events	Subclavian artery thrombosis
Embolic and thrombotic events	Thromboembolectomy
Embolic and thrombotic events	Thrombotic microangiopathy
Embolic and thrombotic events	Thrombotic thrombocytopenic purpura
Embolic and thrombotic events	Transient ischaemic attack
Embolic and thrombotic events	Truncus coeliacus thrombosis
Embolic and thrombotic events	Vascular pseudoaneurysm thrombosis
Embolic and thrombotic events	Vertebral artery occlusion
Embolic and thrombotic events	Vertebral artery thrombosis
Embolic and thrombotic events	Visual acuity reduced transiently
Embolic and thrombotic events	Aseptic cavernous sinus thrombosis
Embolic and thrombotic events	Axillary vein thrombosis
Embolic and thrombotic events	Brachiocephalic vein occlusion
Embolic and thrombotic events	Brachiocephalic vein thrombosis
Embolic and thrombotic events	Budd-Chiari syndrome
Embolic and thrombotic events	Catheterisation venous
Embolic and thrombotic events	Cavernous sinus thrombosis
Embolic and thrombotic events	Central venous catheterisation
Embolic and thrombotic events	Cerebral venous sinus thrombosis
Embolic and thrombotic events	Cerebral venous thrombosis
Embolic and thrombotic events	Compression garment application
Embolic and thrombotic events	Deep vein thrombosis
Embolic and thrombotic events	Deep vein thrombosis postoperative
Embolic and thrombotic events	Embolism venous
Embolic and thrombotic events	Hepatic vein embolism
Embolic and thrombotic events	Hepatic vein occlusion
Embolic and thrombotic events	Hepatic vein thrombosis
Embolic and thrombotic events	Homans' sign positive
Embolic and thrombotic events	Iliac vein occlusion
Embolic and thrombotic events	Inferior vena cava syndrome
Embolic and thrombotic events	Inferior vena caval occlusion
Embolic and thrombotic events	Jugular vein embolism
Embolic and thrombotic events	Jugular vein occlusion
Embolic and thrombotic events	Jugular vein thrombosis
Embolic and thrombotic events	Mahler sign
Embolic and thrombotic events	May-Thurner syndrome
Embolic and thrombotic events	Mesenteric vein thrombosis
Embolic and thrombotic events	Mesenteric venous occlusion
Embolic and thrombotic events	Obstetrical pulmonary embolism
Embolic and thrombotic events	Obstructive shock
Embolic and thrombotic events	Ophthalmic vein thrombosis
Embolic and thrombotic events	Ovarian vein thrombosis
Embolic and thrombotic events	Paget-Schroetter syndrome

Embolic and thrombotic events	Pelvic venous thrombosis
Embolic and thrombotic events	Penile vein thrombosis
Embolic and thrombotic events	Peripheral vein occlusion
Embolic and thrombotic events	Peripheral vein thrombosis
Embolic and thrombotic events	Peripheral vein thrombus extension
Embolic and thrombotic events	Phlebectomy
Embolic and thrombotic events	Portal vein cavernous transformation
Embolic and thrombotic events	Portal vein embolism
Embolic and thrombotic events	Portal vein occlusion
Embolic and thrombotic events	Portal vein thrombosis
Embolic and thrombotic events	Portosplenomesenteric venous thrombosis
Embolic and thrombotic events	Post procedural pulmonary embolism
Embolic and thrombotic events	Post thrombotic syndrome
Embolic and thrombotic events	Postoperative thrombosis
Embolic and thrombotic events	Postpartum venous thrombosis
Embolic and thrombotic events	Pulmonary embolism
Embolic and thrombotic events	Pulmonary infarction
Embolic and thrombotic events	Pulmonary microemboli
Embolic and thrombotic events	Pulmonary thrombosis
Embolic and thrombotic events	Pulmonary vein occlusion
Embolic and thrombotic events	Pulmonary veno-occlusive disease
Embolic and thrombotic events	Pulmonary venous thrombosis
Embolic and thrombotic events	Renal vein embolism
Embolic and thrombotic events	Renal vein occlusion
Embolic and thrombotic events	Renal vein thrombosis
Embolic and thrombotic events	Retinal vein occlusion
Embolic and thrombotic events	Retinal vein thrombosis
Embolic and thrombotic events	SI QIII TIII pattern
Embolic and thrombotic events	Sigmoid sinus thrombosis
Embolic and thrombotic events	Splenic vein occlusion
Embolic and thrombotic events	Splenic vein thrombosis
Embolic and thrombotic events	Subclavian vein occlusion
Embolic and thrombotic events	Subclavian vein thrombosis
Embolic and thrombotic events	Superficial vein thrombosis
Embolic and thrombotic events	Superior sagittal sinus thrombosis
Embolic and thrombotic events	Superior vena cava occlusion
Embolic and thrombotic events	Superior vena cava syndrome
Embolic and thrombotic events	Thrombophlebitis
Embolic and thrombotic events	Thrombophlebitis migrans
Embolic and thrombotic events	Thrombophlebitis neonatal
Embolic and thrombotic events	Thrombosed varicose vein
Embolic and thrombotic events	Thrombosis corpora cavernosa
Embolic and thrombotic events	Transverse sinus thrombosis
Embolic and thrombotic events	Vena cava embolism
Embolic and thrombotic events	Vena cava filter insertion
Embolic and thrombotic events	Vena cava filter removal

Embolic and thrombotic events	Vena cava thrombosis
Embolic and thrombotic events	Venogram abnormal
Embolic and thrombotic events	Venoocclusive disease
Embolic and thrombotic events	Venoocclusive liver disease
Embolic and thrombotic events	Venous angioplasty
Embolic and thrombotic events	Venous occlusion
Embolic and thrombotic events	Venous operation
Embolic and thrombotic events	Venous recanalisation
Embolic and thrombotic events	Venous repair
Embolic and thrombotic events	Venous stent insertion
Embolic and thrombotic events	Venous thrombosis
Embolic and thrombotic events	Venous thrombosis in pregnancy
Embolic and thrombotic events	Venous thrombosis limb
Embolic and thrombotic events	Venous thrombosis neonatal
Embolic and thrombotic events	Visceral venous thrombosis
Embolic and thrombotic events	Administration site thrombosis
Embolic and thrombotic events	Adrenal thrombosis
Embolic and thrombotic events	Angiogram abnormal
Embolic and thrombotic events	Angiogram cerebral abnormal
Embolic and thrombotic events	Angiogram peripheral abnormal
Embolic and thrombotic events	Antiphospholipid syndrome
Embolic and thrombotic events	Application site thrombosis
Embolic and thrombotic events	Arteriovenous fistula occlusion
Embolic and thrombotic events	Arteriovenous fistula thrombosis
Embolic and thrombotic events	Arteriovenous graft thrombosis
Embolic and thrombotic events	Artificial blood vessel occlusion
Embolic and thrombotic events	Atrial thrombosis
Embolic and thrombotic events	Autoimmune heparin-induced thrombocytopenia
Embolic and thrombotic events	Basal ganglia stroke
Embolic and thrombotic events	Bone infarction
Embolic and thrombotic events	Brain stem embolism
Embolic and thrombotic events	Brain stem infarction
Embolic and thrombotic events	Brain stem stroke
Embolic and thrombotic events	Brain stem thrombosis
Embolic and thrombotic events	Cardiac ventricular thrombosis
Embolic and thrombotic events	Catheter directed thrombolysis
Embolic and thrombotic events	Catheter site thrombosis
Embolic and thrombotic events	Cerebellar embolism
Embolic and thrombotic events	Cerebellar infarction
Embolic and thrombotic events	Cerebral congestion
Embolic and thrombotic events	Cerebral infarction
Embolic and thrombotic events	Cerebral infarction foetal
Embolic and thrombotic events	Cerebral ischaemia
Embolic and thrombotic events	Cerebral microembolism
Embolic and thrombotic events	Cerebral microinfarction
Embolic and thrombotic events	Cerebral septic infarct

Embolic and thrombotic events	Cerebral thrombosis
Embolic and thrombotic events	Cerebral vascular occlusion
Embolic and thrombotic events	Cerebrospinal thrombotic tamponade
Embolic and thrombotic events	Cerebrovascular accident
Embolic and thrombotic events	Cerebrovascular accident prophylaxis
Embolic and thrombotic events	Cerebrovascular disorder
Embolic and thrombotic events	Cerebrovascular operation
Embolic and thrombotic events	Choroidal infarction
Embolic and thrombotic events	Collateral circulation
Embolic and thrombotic events	Coronary bypass thrombosis
Embolic and thrombotic events	Device embolisation
Embolic and thrombotic events	Device occlusion
Embolic and thrombotic events	Device related thrombosis
Embolic and thrombotic events	Diplegia
Embolic and thrombotic events	Directional Doppler flow tests abnormal
Embolic and thrombotic events	Disseminated intravascular coagulation
Embolic and thrombotic events	Disseminated intravascular coagulation in newborn
Embolic and thrombotic events	Embolic cerebellar infarction
Embolic and thrombotic events	Embolic cerebral infarction
Embolic and thrombotic events	Embolic pneumonia
Embolic and thrombotic events	Embolic stroke
Embolic and thrombotic events	Embolism
Embolic and thrombotic events	Eye infarction
Embolic and thrombotic events	Fluorescence angiogram abnormal
Embolic and thrombotic events	Foetal cerebrovascular disorder
Embolic and thrombotic events	Foetal vascular malperfusion
Embolic and thrombotic events	Gastric infarction
Embolic and thrombotic events	Graft thrombosis
Embolic and thrombotic events	Haemorrhagic adrenal infarction
Embolic and thrombotic events	Haemorrhagic cerebral infarction
Embolic and thrombotic events	Haemorrhagic infarction
Embolic and thrombotic events	Haemorrhagic stroke
Embolic and thrombotic events	Haemorrhagic transformation stroke
Embolic and thrombotic events	Haemorrhoids thrombosed
Embolic and thrombotic events	Hemiparesis
Embolic and thrombotic events	Hemiplegia
Embolic and thrombotic events	Heparin-induced thrombocytopenia
Embolic and thrombotic events	Hepatic infarction
Embolic and thrombotic events	Hepatic vascular thrombosis
Embolic and thrombotic events	Implant site thrombosis
Embolic and thrombotic events	Incision site vessel occlusion
Embolic and thrombotic events	Infarction
Embolic and thrombotic events	Infusion site thrombosis
Embolic and thrombotic events	Injection site thrombosis
Embolic and thrombotic events	Inner ear infarction

Embolic and thrombotic events	Instillation site thrombosis
Embolic and thrombotic events	Intestinal infarction
Embolic and thrombotic events	Intracardiac mass
Embolic and thrombotic events	Intracardiac thrombus
Embolic and thrombotic events	Lambl's excrescences
Embolic and thrombotic events	Medical device site thrombosis
Embolic and thrombotic events	Mesenteric vascular insufficiency
Embolic and thrombotic events	Mesenteric vascular occlusion
Embolic and thrombotic events	Microembolism
Embolic and thrombotic events	Monoparesis
Embolic and thrombotic events	Monoplegia
Embolic and thrombotic events	Muscle infarction
Embolic and thrombotic events	Optic nerve infarction
Embolic and thrombotic events	Pancreatic infarction
Embolic and thrombotic events	Paradoxical embolism
Embolic and thrombotic events	Paraneoplastic thrombosis
Embolic and thrombotic events	Paraparesis
Embolic and thrombotic events	Paraplegia
Embolic and thrombotic events	Paresis
Embolic and thrombotic events	Peripheral revascularisation
Embolic and thrombotic events	Pituitary infarction
Embolic and thrombotic events	Placental infarction
Embolic and thrombotic events	Pneumatic compression therapy
Embolic and thrombotic events	Portal shunt procedure
Embolic and thrombotic events	Post procedural stroke
Embolic and thrombotic events	Postpartum thrombosis
Embolic and thrombotic events	Prosthetic cardiac valve thrombosis
Embolic and thrombotic events	Prosthetic vessel implantation
Embolic and thrombotic events	Quadriparesis
Embolic and thrombotic events	Quadriplegia
Embolic and thrombotic events	Renal infarct
Embolic and thrombotic events	Renal vascular thrombosis
Embolic and thrombotic events	Retinal infarction
Embolic and thrombotic events	Retinal vascular thrombosis
Embolic and thrombotic events	Revascularisation procedure
Embolic and thrombotic events	Shunt occlusion
Embolic and thrombotic events	Shunt thrombosis
Embolic and thrombotic events	Spinal cord infarction
Embolic and thrombotic events	Spinal stroke
Embolic and thrombotic events	Splenic infarction
Embolic and thrombotic events	Splenic thrombosis
Embolic and thrombotic events	Spontaneous heparin-induced thrombocytopenia syndrome
Embolic and thrombotic events	Stoma site thrombosis
Embolic and thrombotic events	Stroke in evolution
Embolic and thrombotic events	Strokectomy

Embolic and thrombotic events	Surgical vascular shunt
Embolic and thrombotic events	Testicular infarction
Embolic and thrombotic events	Thalamic infarction
Embolic and thrombotic events	Thrombectomy
Embolic and thrombotic events	Thromboangiitis obliterans
Embolic and thrombotic events	Thrombolysis
Embolic and thrombotic events	Thrombosis
Embolic and thrombotic events	Thrombosis in device
Embolic and thrombotic events	Thrombosis mesenteric vessel
Embolic and thrombotic events	Thrombosis prophylaxis
Embolic and thrombotic events	Thrombosis with thrombocytopenia syndrome
Embolic and thrombotic events	Thrombotic cerebral infarction
Embolic and thrombotic events	Thrombotic stroke
Embolic and thrombotic events	Thyroid infarction
Embolic and thrombotic events	Tumour embolism
Embolic and thrombotic events	Tumour thrombectomy
Embolic and thrombotic events	Tumour thrombosis
Embolic and thrombotic events	Ultrasonic angiogram abnormal
Embolic and thrombotic events	Ultrasound Doppler abnormal
Embolic and thrombotic events	Umbilical cord occlusion
Embolic and thrombotic events	Umbilical cord thrombosis
Embolic and thrombotic events	Vaccination site thrombosis
Embolic and thrombotic events	Vascular access site thrombosis
Embolic and thrombotic events	Vascular device occlusion
Embolic and thrombotic events	Vascular graft
Embolic and thrombotic events	Vascular graft occlusion
Embolic and thrombotic events	Vascular graft thrombosis
Embolic and thrombotic events	Vascular operation
Embolic and thrombotic events	Vascular stent insertion
Embolic and thrombotic events	Vascular stent occlusion
Embolic and thrombotic events	Vascular stent thrombosis
Embolic and thrombotic events	Vasodilation procedure
Embolic and thrombotic events	Vessel puncture site occlusion
Embolic and thrombotic events	Vessel puncture site thrombosis
Embolic and thrombotic events	Visual midline shift syndrome
Hypertension	Accelerated hypertension
Hypertension	Blood pressure ambulatory increased
Hypertension	Blood pressure diastolic increased
Hypertension	Blood pressure inadequately controlled
Hypertension	Blood pressure increased
Hypertension	Blood pressure management
Hypertension	Blood pressure orthostatic increased
Hypertension	Blood pressure systolic increased
Hypertension	Catecholamine crisis
Hypertension	Dialysis induced hypertension
Hypertension	Diastolic hypertension

Hypertension	Eclampsia
Hypertension	Endocrine hypertension
Hypertension	Essential hypertension
Hypertension	Gestational hypertension
Hypertension	HELLP syndrome
Hypertension	Hyperaldosteronism
Hypertension	Hypertension
Hypertension	Hypertension neonatal
Hypertension	Hypertensive angiopathy
Hypertension	Hypertensive cardiomegaly
Hypertension	Hypertensive cardiomyopathy
Hypertension	Hypertensive cerebrovascular disease
Hypertension	Hypertensive crisis
Hypertension	Hypertensive emergency
Hypertension	Hypertensive encephalopathy
Hypertension	Hypertensive end-organ damage
Hypertension	Hypertensive heart disease
Hypertension	Hypertensive nephropathy
Hypertension	Hypertensive urgency
Hypertension	Labile hypertension
Hypertension	Malignant hypertension
Hypertension	Malignant hypertensive heart disease
Hypertension	Malignant renal hypertension
Hypertension	Maternal hypertension affecting foetus
Hypertension	Mean arterial pressure increased
Hypertension	Metabolic syndrome
Hypertension	Neurogenic hypertension
Hypertension	Orthostatic hypertension
Hypertension	Page kidney
Hypertension	Postoperative hypertension
Hypertension	Pre-eclampsia
Hypertension	Prehypertension
Hypertension	Procedural hypertension
Hypertension	Renal hypertension
Hypertension	Renal sympathetic nerve ablation
Hypertension	Renovascular hypertension
Hypertension	Retinopathy hypertensive
Hypertension	Secondary aldosteronism
Hypertension	Secondary hypertension
Hypertension	Superimposed pre-eclampsia
Hypertension	Supine hypertension
Hypertension	Systolic hypertension
Hypertension	Withdrawal hypertension
Ischaemic heart disease	Acute cardiac event
Ischaemic heart disease	Acute coronary syndrome
Ischaemic heart disease	Acute myocardial infarction

Ischaemic heart disease	Angina unstable
Ischaemic heart disease	Blood creatine phosphokinase MB abnormal
Ischaemic heart disease	Blood creatine phosphokinase MB increased
Ischaemic heart disease	Coronary artery embolism
Ischaemic heart disease	Coronary artery occlusion
Ischaemic heart disease	Coronary artery reocclusion
Ischaemic heart disease	Coronary artery thrombosis
Ischaemic heart disease	Coronary bypass thrombosis
Ischaemic heart disease	Coronary vascular graft occlusion
Ischaemic heart disease	Kounis syndrome
Ischaemic heart disease	Myocardial infarction
Ischaemic heart disease	Myocardial necrosis
Ischaemic heart disease	Myocardial reperfusion injury
Ischaemic heart disease	Myocardial stunning
Ischaemic heart disease	Papillary muscle infarction
Ischaemic heart disease	Periprocedural myocardial infarction
Ischaemic heart disease	Post procedural myocardial infarction
Ischaemic heart disease	Postinfarction angina
Ischaemic heart disease	Silent myocardial infarction
Ischaemic heart disease	Troponin I increased
Ischaemic heart disease	Troponin increased
Ischaemic heart disease	Troponin T increased
Ischaemic heart disease	Acute cardiac event
Ischaemic heart disease	Angina pectoris
Ischaemic heart disease	Angina unstable
Ischaemic heart disease	Anginal equivalent
Ischaemic heart disease	Arteriosclerosis coronary artery
Ischaemic heart disease	Arteriospasm coronary
Ischaemic heart disease	Cardiac perfusion defect
Ischaemic heart disease	Chronic coronary syndrome
Ischaemic heart disease	Coronary angioplasty
Ischaemic heart disease	Coronary arterial stent insertion
Ischaemic heart disease	Coronary artery bypass
Ischaemic heart disease	Coronary artery compression
Ischaemic heart disease	Coronary artery disease
Ischaemic heart disease	Coronary artery dissection
Ischaemic heart disease	Coronary artery insufficiency
Ischaemic heart disease	Coronary artery restenosis
Ischaemic heart disease	Coronary artery stenosis
Ischaemic heart disease	Coronary artery surgery
Ischaemic heart disease	Coronary brachytherapy
Ischaemic heart disease	Coronary bypass stenosis
Ischaemic heart disease	Coronary endarterectomy
Ischaemic heart disease	Coronary no-reflow phenomenon
Ischaemic heart disease	Coronary ostial stenosis
Ischaemic heart disease	Coronary revascularisation

Ischaemic heart disease	Coronary steal syndrome
Ischaemic heart disease	Coronary vascular graft stenosis
Ischaemic heart disease	Diabetic coronary microangiopathy
Ischaemic heart disease	ECG signs of myocardial ischaemia
Ischaemic heart disease	External counterpulsation
Ischaemic heart disease	Haemorrhage coronary artery
Ischaemic heart disease	Ischaemic cardiomyopathy
Ischaemic heart disease	Ischaemic mitral regurgitation
Ischaemic heart disease	Microvascular coronary artery disease
Ischaemic heart disease	Myocardial hypoperfusion
Ischaemic heart disease	Myocardial hypoxia
Ischaemic heart disease	Myocardial ischaemia
Ischaemic heart disease	Percutaneous coronary intervention
Ischaemic heart disease	Prinzmetal angina
Ischaemic heart disease	Stress cardiomyopathy
Ischaemic heart disease	Subclavian coronary steal syndrome
Ischaemic heart disease	Subendocardial ischaemia
Ischaemic heart disease	Wellens' syndrome
Pulmonary hypertension	Acute right ventricular failure
Pulmonary hypertension	Cardiac ventriculogram right abnormal
Pulmonary hypertension	Central venous pressure increased
Pulmonary hypertension	Chronic right ventricular failure
Pulmonary hypertension	Cor pulmonale
Pulmonary hypertension	Cor pulmonale acute
Pulmonary hypertension	Cor pulmonale chronic
Pulmonary hypertension	Coronary sinus dilatation
Pulmonary hypertension	Portopulmonary hypertension
Pulmonary hypertension	Pulmonary arterial hypertension
Pulmonary hypertension	Pulmonary arterial pressure abnormal
Pulmonary hypertension	Pulmonary arterial pressure increased
Pulmonary hypertension	Pulmonary arterial wedge pressure increased
Pulmonary hypertension	Pulmonary artery dilatation
Pulmonary hypertension	Pulmonary artery wall hypertrophy
Pulmonary hypertension	Pulmonary capillary haemangiomatosis
Pulmonary hypertension	Pulmonary endarterectomy
Pulmonary hypertension	Pulmonary hypertension
Pulmonary hypertension	Pulmonary hypertensive crisis
Pulmonary hypertension	Pulmonary tumour thrombotic microangiopathy
Pulmonary hypertension	Pulmonary valve incompetence
Pulmonary hypertension	Pulmonary vascular resistance abnormality
Pulmonary hypertension	Pulmonary vein occlusion
Pulmonary hypertension	Pulmonary vein stenosis
Pulmonary hypertension	Pulmonary veno-occlusive disease
Pulmonary hypertension	Pulmonary venous hypertension
Pulmonary hypertension	Right atrial dilatation
Pulmonary hypertension	Right atrial enlargement

Pulmonary hypertension	Right atrial hypertrophy
Pulmonary hypertension	Right atrial pressure increased
Pulmonary hypertension	Right atrial volume increased
Pulmonary hypertension	Right ventricular dilatation
Pulmonary hypertension	Right ventricular dysfunction
Pulmonary hypertension	Right ventricular ejection fraction decreased
Pulmonary hypertension	Right ventricular enlargement
Pulmonary hypertension	Right ventricular failure
Pulmonary hypertension	Right ventricular heave
Pulmonary hypertension	Right ventricular hypertension
Pulmonary hypertension	Right ventricular hypertrophy
Pulmonary hypertension	Right ventricular systolic pressure increased
Pulmonary hypertension	Tricuspid valve incompetence
Pulmonary hypertension	Vascular resistance pulmonary increased
Torsade de pointes/QT prolongation	Electrocardiogram QT interval abnormal
Torsade de pointes/QT prolongation	Electrocardiogram QT prolonged
Torsade de pointes/QT prolongation	Long QT syndrome
Torsade de pointes/QT prolongation	Long QT syndrome congenital
Torsade de pointes/QT prolongation	Torsade de pointes
Torsade de pointes/QT prolongation	Ventricular tachycardia

Supplementary Table S3. Formulas used to calculate Reporting Odds Ratio (ROR) and Information Component (IC) with their 95% confidence interval (CI) for disproportionality analysis.

	Drug of interests	Other drugs
Adverse events of interests	a	b
Other adverse events	c	d

$$ROR = \frac{ad}{bc}$$

$$95\%\ \text{CI} = e^{\ln(ROR) \pm 1.96\sqrt{(\frac{1}{a}+\frac{1}{b}+\frac{1}{c}+\frac{1}{d})}}$$

$$IC = \log_2 \frac{a+0.5}{\frac{(a+b)(a+c)}{a+b+c+d}+0.5}$$

Lower 95% CI = $IC - 3.3 \times (a + 0.5)^{-\frac{1}{2}} - 2 \times (a + 0.5)^{-\frac{3}{2}}$

Upper 95% CI = $IC + 2.4 \times (a + 0.5)^{-\frac{1}{2}} - 0.5 \times (a + 0.5)^{-\frac{3}{2}}$

References:

Van Puijenbroek, E.P.; Bate, A.; Leufkens, H.G.; Lindquist, M.; Orre, R.; Egberts, A.C. A comparison of measures of disproportionality for signal detection in spontaneous reporting systems for adverse drug reactions. *Pharmacoepidemiol. Drug Saf.* **2002**, *11*, 3–10. https://doi.org/10.1002/pds.668

Bate, A.; Lindquist, M.; Edwards, I.R.; Olsson, S.; Orre, R.; Lansner, A.; De Freitas, R.M. A Bayesian neural network method for adverse drug reaction signal generation. *Eur. J. Clin. Pharmacol.* **1998**, *54*, 315–321. https://doi.org/10.1007/s002280050466

Noren, G.N.; Hopstadius, J.; Bate, A. Shrinkage observed-to-expected ratios for robust and transparent large-scale pattern discovery. *Stat. Methods Med. Res.* **2011**, *22*, 57-69. https://doi.org/10.1177/0962280211403604

Supplementary Figure S1. Sensitivity analysis: Forest plot of disproportionality analysis of cardiovascular adverse events (CVAE) for monoclonal antibody products for COVID-19, by only including CVAE reported by healthcare professionals.

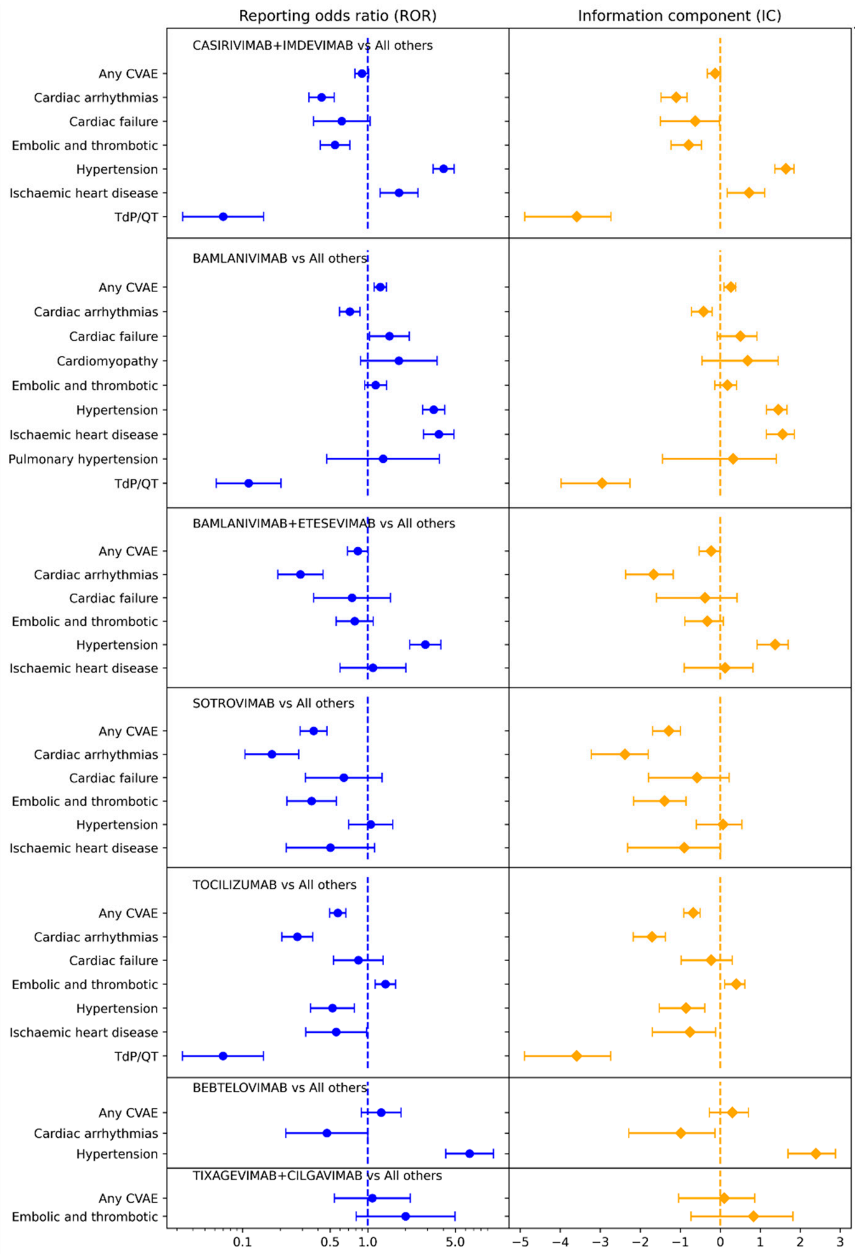

		ROR (95% CI)	IC (95% CI)
CASIRIVIMAB+IMDEVIMAB vs All others	Any CVAE	0.897 (0.790, 1.019)	-0.128 (-0.323, 0.013)
	Cardiac arrhythmias	0.428 (0.339, 0.540)	-1.102 (-1.482, -0.828)
	Cardiac failure	0.620 (0.368, 1.044)	-0.623 (-1.494, -0.022)
	Embolic and thrombotic	0.547 (0.417, 0.718)	-0.788 (-1.232, -0.470)
	Hypertension	4.026 (3.317, 4.887)	1.642 (1.366, 1.842)
	Ischaemic heart disease	1.775 (1.253, 2.515)	0.721 (0.173, 1.111)
	TdP/QT	0.070 (0.033, 0.147)	-3.585 (-4.887, -2.733)
BAMLANIVIMAB vs All others	Any CVAE	1.258 (1.122, 1.411)	0.265 (0.093, 0.389)
	Cardiac arrhythmias	0.718 (0.595, 0.867)	-0.420 (-0.720, -0.202)
	Cardiac failure	1.487 (1.033, 2.141)	0.503 (-0.077, 0.915)
	Cardiomyopathy	1.769 (0.876, 3.571)	0.686 (-0.453, 1.447)
	Embolic and thrombotic	1.155 (0.945, 1.411)	0.182 (-0.135, 0.411)
	Hypertension	3.355 (2.738, 4.110)	1.452 (1.155, 1.667)
	Ischaemic heart disease	3.687 (2.788, 4.874)	1.562 (1.154, 1.856)
	Pulmonary hypertension	1.324 (0.470, 3.727)	0.322 (-1.443, 1.401)
	TdP/QT	0.112 (0.062, 0.203)	-2.953 (-3.978, -2.258)
BAMLANIVIMAB+ETESEVIMAB vs All others	Any CVAE	0.832 (0.688, 1.007)	-0.228 (-0.529, -0.011)
	Cardiac arrhythmias	0.290 (0.191, 0.439)	-1.665 (-2.363, -1.174)
	Cardiac failure	0.749 (0.370, 1.515)	-0.381 (-1.593, 0.422)
	Embolic and thrombotic	0.786 (0.559, 1.105)	-0.322 (-0.885, 0.079)
	Hypertension	2.880 (2.163, 3.834)	1.372 (0.920, 1.696)
	Ischaemic heart disease	1.098 (0.599, 2.013)	0.123 (-0.901, 0.818)
SOTROVIMAB vs All others	Any CVAE	0.369 (0.288, 0.473)	-1.285 (-1.690, -0.994)
	Cardiac arrhythmias	0.171 (0.104, 0.281)	-2.383 (-3.225, -1.800)
	Cardiac failure	0.643 (0.318, 1.301)	-0.582 (-1.794, 0.221)
	Embolic and thrombotic	0.356 (0.226, 0.562)	-1.393 (-2.164, -0.856)
	Hypertension	1.055 (0.704, 1.581)	0.071 (-0.598, 0.543)
	Ischaemic heart disease	0.503 (0.224, 1.132)	-0.900 (-2.315, 0.011)
TOCILIZUMAB vs All others	Any CVAE	0.576 (0.496, 0.668)	-0.675 (-0.911, -0.504)
	Cardiac arrhythmias	0.274 (0.206, 0.363)	-1.706 (-2.176, -1.370)
	Cardiac failure	0.840 (0.533, 1.326)	-0.225 (-0.976, 0.300)
	Embolic and thrombotic	1.381 (1.146, 1.665)	0.404 (0.112, 0.614)
	Hypertension	0.523 (0.349, 0.782)	-0.855 (-1.524, -0.384)
	Ischaemic heart disease	0.559 (0.320, 0.978)	-0.758 (-1.697, -0.115)
	TdP/QT	0.070 (0.033, 0.147)	-3.590 (-4.892, -2.738)
BEBTELOVIMAB vs All others	Any CVAE	1.278 (0.888, 1.839)	0.302 (-0.270, 0.708)
	Cardiac arrhythmias	0.471 (0.222, 1.000)	-0.985 (-2.287, -0.133)
	Hypertension	6.497 (4.193, 10.066)	2.390 (1.692, 2.881)
TIXAGEVIMAB+CILGAVIMAB vs All others	Any CVAE	1.086 (0.540, 2.183)	0.099 (-1.040, 0.861)
	Embolic and thrombotic	2.003 (0.807, 4.974)	0.832 (-0.730, 1.817)

For each type of CVAE, only those with at least 4 reports were analyzed and presented. A significant safety signal is defined as the 95% confidence interval (CI) for ROR is on the right side of the vertical reference line in blue (indicating lower bound of 95% CI is greater than 1) and the 95% CI for IC is on the right side of the vertical reference line in orange (indicating lower bound of 95% CI is greater than 0).
